# Modified Chaotic Hénon Map-Based Text Information Encryption and Hiding Mechanism Using Bottlenose Dolphin Vocalizations

**DOI:** 10.3390/s26082541

**Published:** 2026-04-20

**Authors:** Chin-Feng Lin, Ching-Lung Hsieh, Shun-Hsyung Chang, Ivan A. Parinov, Sergey Shevtsov

**Affiliations:** 1 Department of Electrical Engineering, National Taiwan Ocean University, Keelung 20224, Taiwan; 21153004@mail.ntou.edu.tw; 2 Department of Microelectronics Engineering, National Kaohsiung University of Science and Technology, Kaohsiung 81157, Taiwan; 3 I. I. Vorovich Mathematics, Mechanics, and Computer Science Institute, Southern Federal University, 344090 Rostov-on-Don, Russia; iparinov@sfedu.ru; 4 Head of Aircraft Systems and Technologies Lab at the South Center of Russian Academy of Science, 344006 Rostov-on-Don, Russia; shevtsov@ssc-ras.ru

**Keywords:** modified chaotic Hénon map, text information, hiding, encryption, bottlenose dolphin vocalizations

## Abstract

As ocean resources are further developed and utilized, bionic covert underwater acoustic communication (CUAC) is increasingly important for military and underwater telemetry applications. The primary purpose of this study was to design a highly secure and undetectable text information (TI) encryption mechanism to realize CUAC using real bottlenose dolphin vocalizations (BDVs). For this purpose, a chaotic encryption scheme, spread spectrum (SS) technology, and a modified chaotic Hénon map (MCHM) were integrated into a TI encryption and hiding (EH) mechanism. Four BDVs and four test TIs were employed to demonstrate the performance of the proposed MCHM-based TI EH mechanism (MCHMTIEHM). The simulation results show that the MCHMTIEHM yields more accurate de-hiding and decryption results. When the correct encryption and decryption parameters were used, the test TI was completely recovered and could be recognized by humans. When the MCHM encryption and decryption parameters ${SP}_{x}\;and\;n_{I}\;$ were not identical, tests involving TI01, TI02, TI03, and TI04 demonstrated correct de-hiding and error decryption performance; in particular, the test TI had superior correct de-hiding and error decryption results, was unrecoverable, and could not be recognized by the human eye. The modified amplitude correlation coefficient (ACC) and modified unified average amplitude change intensity (UACI) metrics were used to evaluate the hiding performance of MCHM-based encryption of TI using BDVs. The simulation results show that the average modified ACC and average UACI were 0.99995924 and $3.84\times 10^{-6}$, respectively. Performance was evaluated in terms of the average number of changing SS bit rates (NCSSBRs), the average number of changing bit rates (NCBRs), and the average number of changing character rates (NCCRs) for correct de-hiding and correct/erroneous TI decryption. The average NCSSBRs, NCBRs, and NCCRs were all 0% in correct de-hiding and decryption scenarios, while they were 49.29%, 47.65%, and 98.10%, respectively. with correct de-hiding and error-decryption scenarios. In summary, the proposed MCHMTIEHM yields superior encryption and hiding performance.

## 1. Introduction

Information hiding (IH), originating from acrostics and steganography, has become an important research field. IH can be applied to digital copyright, cloud-based, and military communications systems [1]. Petitcols et al. [1] provided a survey of IH, detailing what we know about it, what works, and what does not. At its core, IH is about making one’s existence covert, i.e., hiding messages in other media, such as text, audio, images, and video. IH technologies include covert communication (CC), steganography, and digital watermarking. Reversible data-hiding schemes involve plain and encryption domains [2]. In the plain domain, secret information is embedded in covert signals without encryption, whereas in the encryption domain, covert signals are encrypted prior to embedding to achieve greater privacy. Covert signals can include text, images, audio, video, medical, and multimedia signals. In addition, an embedded space is created for the secret information carried in the covert signals. As it is highly secure, CC has potential for application in cloud-based systems [3]. The hiding mechanism uses a transmitter that embeds secret messages in environmental or artificial noise, thereby avoiding detection by hackers. Encryption-based cryptography (CRY) provides advantages in terms of authorization, and it is unlikely that data subjected to IH will be detected/intercepted during transmission [4]. The utility of physical-layer CC techniques over additive white Gaussian noise—such as spread spectrum (SS)—has been demonstrated, with CC successfully implemented in Internet of Things (IoT) scenarios, enabling a sufficiently high degree of information secrecy. Sixth-generation (6G) non-terrestrial/seamless communication technologies can be utilized to enhance the potential of stringent IoT services [5]. Mobile ubiquitous CC can conceal the presence of transmitted messages to reduce potential attack threats and is a safety-critical method for resisting security threats.

Direct sequence spread spectrum (DSSS) technology is used in multiple access transmission systems to provide high security, a noise-like appearance, and reliable data transfer [6]. DSSS-based hiding of information in audio signals has been investigated and evaluated in various studies, aiming to conceal data by converting meaningful and significant information into noise-like forms. Steganographic IH technologies are effective solutions for commercial applications, such as copyright protection and digital watermarking. It is difficult to detect hidden information without the corresponding recovery keys. The security aspect of IH is attracting increasing attention, especially among parties interested in providing private IoT transmission services and in preventing interception [7].

The embedding space, embedding process, and reducing and smoothing approaches have attracted significant attention for data hiding in audio signals. In this context, the use of transmission approaches based on embedding in ocean noise can increase information security. Huang et al. [8] proposed a covert underwater acoustic communication (CUAC) scheme using ship-radiated noise and a chaotic Chebyshev signal. Mimic ship-radiated noise carrying secret information was generated by combining the time and frequency characteristics of the chaotic signal and the ship-radiated noise. As ship-radiated noise spans a wide spectral range, it is difficult for an adversary to detect. Therefore, with this scheme, strong robustness and high undetectability can be achieved.

Farschi et al. [9] proposed a digital image steganographic method based on a chaotic dynamical system, which offers low time complexity, a large key space, and high security. Chaos-based hiding systems are characterized by sensitivity to initial parameters, pseudo-random behavior, and continuous broadband signal spectra. Yavuz [10] proposed a chaotic image steganography approach integrating the Chinese Remainder Theorem for data embedding. Through this design, a balance between security, information recovery, and computational efficiency was achieved for the chaotic IH approach. The hidden information can be recovered if both the chaotic maps and the IH keys are available, and the proposed scheme applies to cloud storage scenarios. Lin et al. [11] demonstrated several basic system design concepts for chaos-based medical image encryption, exploring multiple chaotic maps and robust, fast, and simple chaotic encryption mechanisms. Chaos-based encryption methods are characterized by their sensitive dependence on the initial system parameters, seemingly random trajectories, highly unpredictable and complex behaviors, and a lack of periodicity. The authors concluded that chaotic encryption algorithms offer significant advantages for real-time CRY of medical images.

A method for covert acoustic encryption and transmission to achieve IH among IoT devices at the physical layer using random noise has been developed to address privacy issues [12]. The wave shape and noise of the signals can be generated using speakers and microphones, and hidden domains can be created to embed messages. The encryption and IH capacity are stronger than the eavesdropper’s ability to extract information, making the information undetectable for eavesdroppers. A multiple-carrier index-keying orthogonal frequency-division multiplexing-based covert (low-probability detection; LPD) communication technology integrating a chaotic modulation algorithm has been developed to enhance data covertness and achieve effective concealment [13]. An adversarial machine-learning-based perturbation method was used to mitigate an attacker’s ability to intercept covert information. Hybrid chaotic covert communication systems have also been developed; for example, a CUAC scheme using DSSS (m-sequence) and a coherent RAKE receiver has been proposed to achieve a low probability of detection [14]. The time-varying characteristics of the underwater acoustic channel and frequency-selective fading channel with long-term memory were investigated, and a single-user application scenario was designed.

As an example of an approach using biological mimicry, dolphin clicks have been proposed as information carriers for CUAC [15]. In particular, the message bits are conveyed in the time interval between intrinsic dolphin clicks, using pulse-position modulation to achieve a low probability of interception in very shallow water environments. Matching-pursuit-based channel estimation and adaptive RAKE equalization were utilized for the receiver. Kim et al. [16] presented a biomimetic underwater acoustic communication (UAC) system with high covertness and a high data transmission rate. This combines the mimicking of dolphin group whistles, time/frequency-shift keying modulation, and continuous-varying carrier frequency modulation. Dolphin and whale vocalizations have a wide bandwidth, which makes them suitable for increasing covertness and decreasing the probability of detection in CUAC scenarios. An interleaver scheme can be used to mitigate the effects of interference on the whistles. Bionic CC plays an important role in meeting the requirements for the secure transmission and concealment of underwater information, enabling a low probability of UAC recognition [17]. The time/frequency contours of bottlenose dolphin whistles have been adopted to achieve robust steganography, and a virtual time-reversal mirror equalization scheme was designed to reduce underwater multipath fading. A biological CUAC (BCUAC) modem using dolphin whistle and click vocalizations has been designed for use in various covert scenarios [18], in which the message bits are conveyed during the time interval between dolphin clicks. The BCUAC modem can be applied in underwater uncrewed vehicles, remotely operated vehicles, and underwater wireless sensor networks. Furthermore, BCUAC technology has been used to decrease the power spectral density of the transmission information signal, spreading the message energy over a wide bandwidth to make it appear similar to background noise [19]. The various contours of real dolphin whistles are used to confuse transmission signals with underwater dolphin vocalizations, enabling high-degree mimicry. Machine-learning-based whistle detectors were integrated into the BCUAC system, and the system achieved a superior transmission bit-error rate of 0.002.

The chaotic Hénon map [20,21] is a two-dimensional (2D) iteration equation with quadratic nonlinearity and strange attractors, and a chaotic Hénon-based exclusive or (XOR) operation can be applied to generate confusion and diffusion in audio encryption signals. The chaotic Hénon map is a dynamical nonlinear system that can be used to generate pseudo-random time series data, which is characterized by ergodicity, randomness, unpredictability, and sensitivity to initial and control parameters. The encryption time series of a chaotic Hénon system can be decrypted if the decryption parameters are known; otherwise, the signal appears as random. CRY, steganography, and watermarking are common techniques for ensuring that sensitive messages are sent securely [22]. A novel audio CRY system based on a substitution–permutation algorithm has been proposed, which uses a Mobius transformation to generate strong 8 × 8 S-boxes for substitution. Meanwhile, a Hénon chaotic map is integrated to permute the positions of the audio data without changing the amplitude values of the original audio signal. Notably, the authors demonstrated that this security solution can transmit detail-scarce images in real time over a wireless channel using chaotic diffusion and confusion operations [23]. Here, the XOR-based diffusion and confusion operations are both generated based on 2D Hénon and Baker chaotic maps. The validity and robustness of the proposed encryption algorithm were analyzed, and it proved to be suitable for resisting various attacks.

Jones et al. [24] reviewed the literature and provided spectrograms and relative time domain waveforms of bottlenose dolphin vocalizations (BDVs), including whistles, squeals, buzzes, barks, quacks, and pops. The bandwidth of sounds that humans can hear is less than 20 kHz, while the bandwidth of BDVs is at least 150 kHz. Bi-phonation-subtype whistle-squawks of the BDVs involved simultaneous whistle-and-burst pulse vocalization, with a frequency range spanning from 0 to 100 kHz. Interactions between fishing activities and dolphins have negative effects on both bottlenose dolphins and fishermen [25]. In one study, whistle, click, and pulse acoustic vocalizations were collected in the context of interactions between common bottlenose dolphins (*Tursiops truncatus*) and fishing activities in the Adriatic Sea. The three main types of BDVs were frequency-modulated whistles, echolocation clicks, and multiple-burst-pulse signals. The bandwidth of the whistle BDVs was reported to range from 0 to 80 kHz. The authors of [26] proposed the interleaver-based modified chaotic logistic-sine (MCLS) TI encryption and hiding (EH) mechanism (MCLSTIEHM), which included the technical characteristics of BDVs and an 8 × 8 interleaver. This approach enables the secure, robust transmission of text information and can be applied to CUAC and multimedia signal applications. A part of this article has been previously published in the Conference on 2025 COUTA and 26 UT [27].

The remainder of this paper is organized as follows. Section 2 presents the modified chaotic Hénon map (MCHM)-based TI EH mechanisms using BDVs. Section 3 details the concealment, correct decryption, and error decryption performance of the proposed approach, while Section 4 presents its performance regarding the average modified amplitude correlation coefficients (ACCs) and average modified unified average amplitude change intensities (UACIs) for TI hiding. Section 5 explores performance metrics for the average number of changing SS bit rates (NCSSBRs), the number of changing bit rates (NCBRs), and the number of changing character rates (NCCRs) for correct and erroneous TI decryptions. Finally, the discussion and concluding remarks are presented in Section 6 and Section 7, respectively.

## 2. MCHM-Based TI EH Mechanism Using BDVs

**Figure 1:**
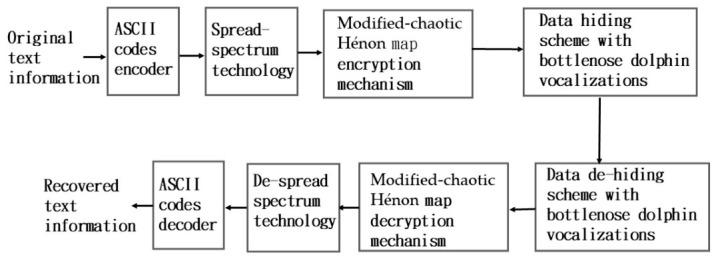
Proposed MCHM-based TI EH mechanism using BDVs.

Figure 1 shows the framework of the proposed MCHM-based TI EH mechanism (MCHMTIEHM) using BDVs, enabling TI to be encrypted and embedded in CUAC applications. This scheme integrates an American Standard Code for Information Interchange (ASCII) code encoder, SS technology, an MCHM encryption mechanism, and a data-hiding scheme (DHS) utilizing BDV signals.

The original TI is used as input to the ASCII code encoder, and the original TI’s ASCII bit streams are extracted as output. The original TI ASCII bit streams then serve as an input for the SS technology, and the original TI ASCII SS bit streams are extracted as the output. The original TI ASCII SS bit streams are then used as an input for the MCHM encryption mechanism (MCHMEM), and the original TI ASCII SS MCHMEM signals are extracted as the output. Finally, the original TI ASCII SS MCHMEM signals serve as an input for the DHS with BDVs, and the BDVs, including the hidden TI, are extracted as the output.

The MCHMEM, which oscillates in 2D space, is a nonlinear discrete-time dynamical system given by Equation (1):

(1) $$x_{n+1}=1-\alpha x_{n}^{2}+y_{n}$$

$$y_{n+1}=\beta x_{n}$$

where x and y are the iterated values, n=0,1,2,3, ⋯ denotes the number of iterations, and α and β are the two bifurcation control parameters of the MCHM (with values of 1.4 and 0.3, respectively, used in the illustrative examples in this paper). The CRY parameters, consisting of the number of discarded initial MCHM index points ($n_{I}$) and the number of security levels ($\delta_{SL})$, are set to enhance the robustness and unpredictability of CRY.

**Figure 2:**
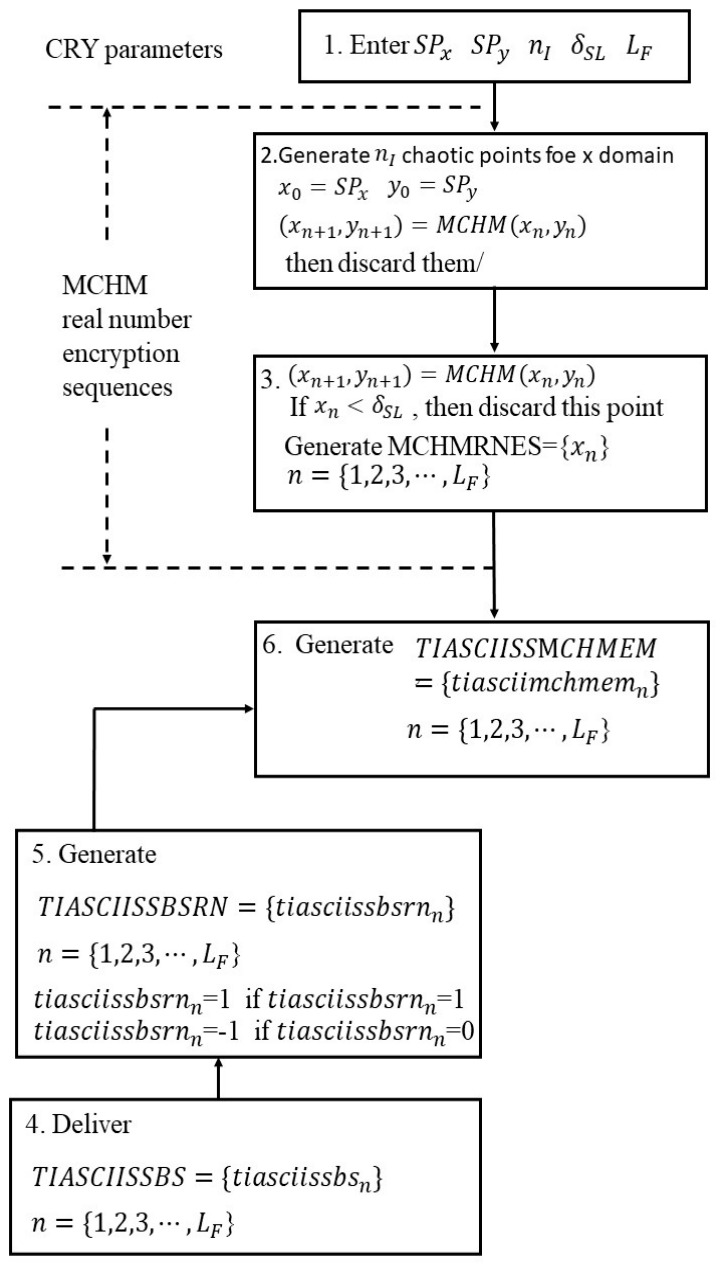
Flowchart of the proposed MCHMEM.

Figure 2 displays a flow chart of the MCHMEM, which is described below.

Step 1: Enter the starting points ${SP}_{x}$ and ${SP}_{y}$, bifurcation control parameters α and β, the length $L_{F}$ of the MCHM real-number encryption sequence for the x domain, the number of discarded initial MCHM index points $n_{I}$, and the number of security levels $\delta_{SL}$.
Step 2: (a) $x_{0}={SP}_{x}$, $y_{0}={SP}_{y}$;

(b) Generate $n_{I}$ chaotic points for the x domain.

(2) $$(x_{n+1,}\;y_{n+1})=\mathrm{MCHM}(x_{n,}\;y_{n})$$

Then, discard them.

Step 3: (a) ($x_{nI+1,}\;y_{nI+1})$ = MCHM($x_{nI,}\;y_{nI})$

(b) If $x_{n}$ < $\delta_{SL}$, then discard this point and go to step 3(a);

otherwise, perform step 3(c);

(c) Generate MCHM real-number encryption sequences, MCHMRNES, with a length of $L_{F}.$

MCHMRNES = {***x_n_***}, n = {1, 2, 3,⋯, ***L_F_***} (3)

Step 4: Deliver the original TI ASCII SS bit streams, TIASCIISSBS, with a length of $L_{F}.$
  TIASCIISSBS = {***tiasciissbs_n_***}, n = {1, 2, 3, ⋯, ***L_F_***} (4)
Step 5: Generate *TIASCIISSBSRN*:
  TIASCIISSBSRN = {***tiasciissbsrn_n_***}, n = {1, 2, 3, ⋯, ***L_F_***} (5)
  $${tiasciissbsrn}_{n}=1\;\;\;\;\mathrm{if}\;\;\;\;{tiasciissbs}_{n}=1$$
  $${tiasciissbsrn}_{n}=-1\;\;\;\;\mathrm{if}\;\;\;\;{tiasciissbs}_{n}=0$$
Step 6: Generate the original TI ASCII SS MCHMEM signals: *TIASCIISS MCHMEM*
  *TIASCIISSMCHMEM* = MCHMRNES × TIASCIISSBSRN            = {***tiasciimchmem_n_***} n = {1, 2, 3, ⋯, ***L_F_***} (6)
  $${tiasciimchmem}_{n}=x_{n}\times {tiasciissbsm}_{n}$$

**Figure 3:**
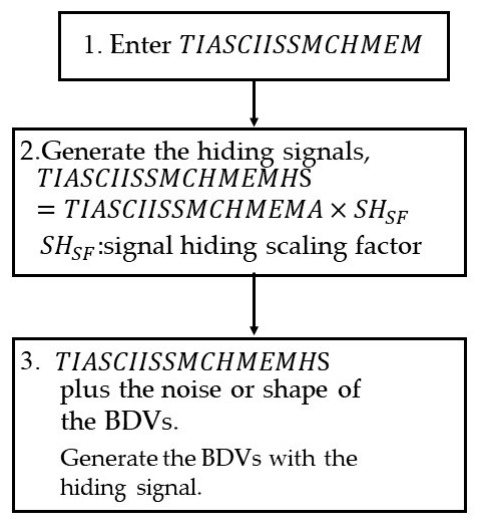
Flowchart of the proposed DHS.

Figure 3 shows a flowchart of the DHS, which is described below.

Step 1: Enter *TIASCIISSMCHMEM*.
Step 2: Generate the hiding signals, *TIASCIISSMCHMEMHS*
  *TIASCIISSMCHMEMHS* = *TIASCIISSMCHMEM* × ***SH_SF_***                = {***tiasciimchmemsh_n_***} n = {1, 2, 3, ⋯, ***L_F_***} (7)
  $${tiasciimchmemsh}_{n}={tiasciimchmem}_{n}\times {SH}_{SF}$$
  $${SH}_{SF}:signal-hiding\;scaling\;factor$$
Step 3: *TIASCIISSMCHMEMHS* plus the noise or shape of the BDVs.

Generate the BDVs with the hiding signal.

The data de-hiding and TI-decryption mechanisms are the inverse functions of the DHS and the MCHMEM, respectively.

## 3. Concealment, Correct Decryption, and Error Decryption Performance

**Figure 4:**
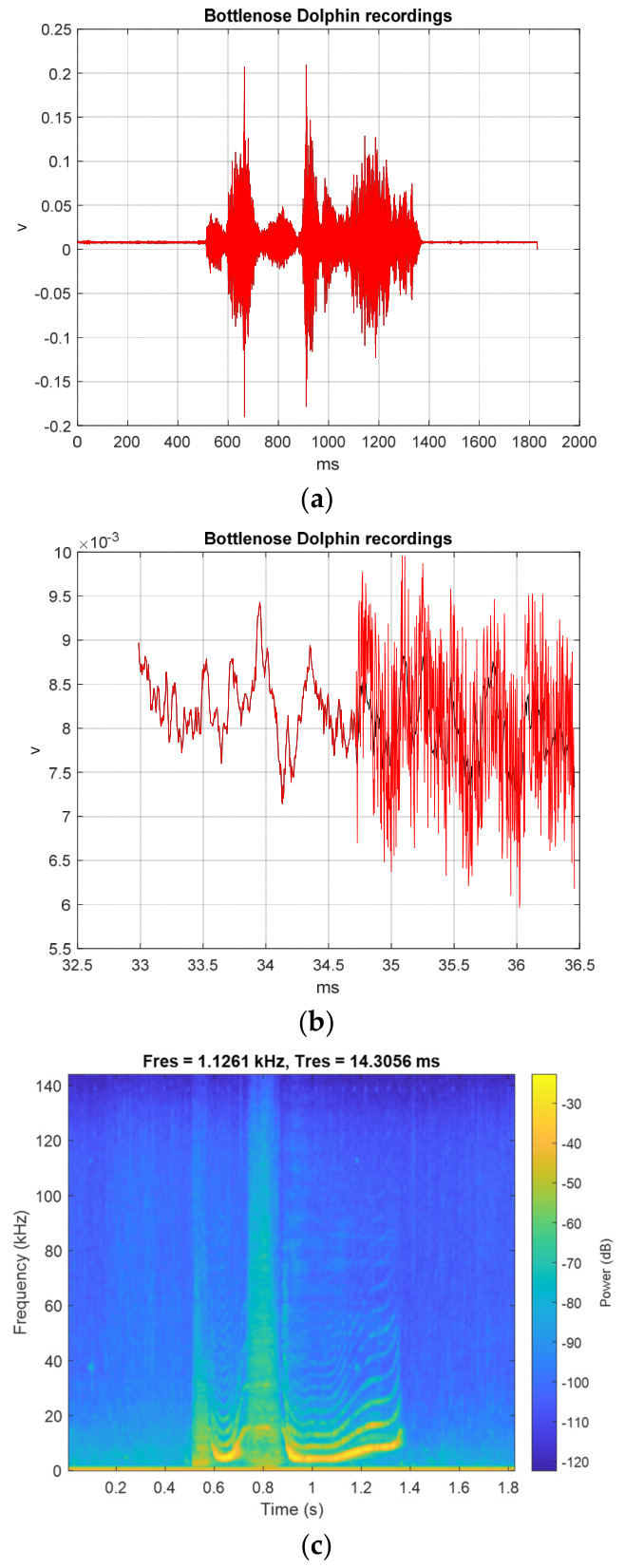

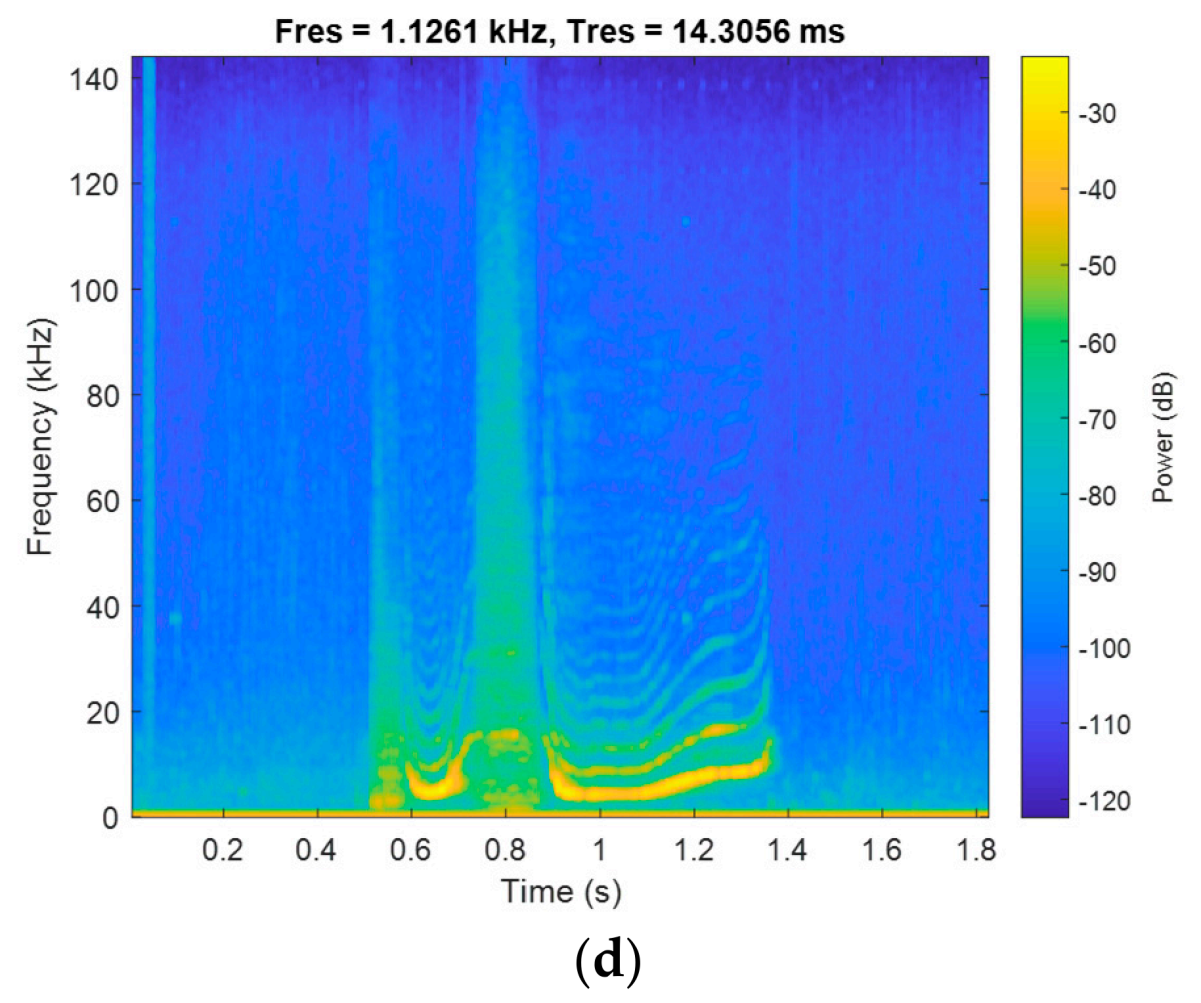
BDV (tbio_a_1613265_sm9609) processed with the MCHMTIEHM: (**a**) the time interval from 0 to 1831 ms; (**b**) the time interval from 32.99 to 36.40 ms; (**c**) the spectrogram of original BDV (tbio_a_1613265_sm9609); (**d**) the spectrogram of BDV (tbio_a_1613265_sm9609) hiding the encrypted signal. Black line, original BDV; red line, BDV hiding the encrypted signal.

The original BDVs (tbio_a_1613265_sm9609 and tbio_a_1613265_sm9612) were downloaded from the following source: https://figshare.com/articles/dataset/Sounds_produced_by_bottlenose_dolphins_i_Tursiops_i_a_review_of_the_defining_characteristics_and_acoustic_criteria_of_the_dolphin_vocal_repertoire/12853107 (accessed on 26 January 2026) [24,28]. Another original BDV (006_whistle) was downloaded from https://figshare.com/collections/Bottlenose_dolphin_s_Tursiops_truncatus_Montagu_1821_acoustic_emissions_recorded_during_interaction_with_bottom_trawl_nets_in_thecentral-northern_Adriatic_Sea/6313308 (accessed on 26 January 2026) [25,29]. The original TI01 text was ‘Chaotic Hénon Map-based TI Encryption and Hiding Mechanisms’, with the following MCHM encryption parameters—${\alpha ,\beta ,SP}_{x},{SP}_{y},\delta_{SL},\;and\;n_{I}$—set as 1.4, 0.3, 0.63, 0.19, 0.1, and 10,000, respectively, while the spread factor (*SF*) of SS for the original ASCII TI bit streams was 9. Figure 4a shows the BDV (tbio_a_1613265_sm9609) processed with the MCHMTIEHM in the time interval from 0 ms to 1831 ms. The TI01 MCHM encryption signal was embedded into the noise of the BDV (tbio_a_1613265_sm9609) in the time interval from 32.99 to 36.40 ms, as shown in Figure 4b. The black line represents the original BDV, whereas the red line represents the BDV hiding the encrypted signal. Figure 4c shows the spectrogram of the original BDV (tbio_a_1613265_sm9609) in the time interval 0 to 1831 ms. Figure 4d shows the spectrogram of BDV (tbio_a_1613265_sm9609) hiding the encrypted signal in the time interval 0 to 1831 ms. Figure 4c,d are approximately the same, and the proposed MCHMTIEHM yields superior hiding performance in the spectrogram domain.

**Figure 5:**
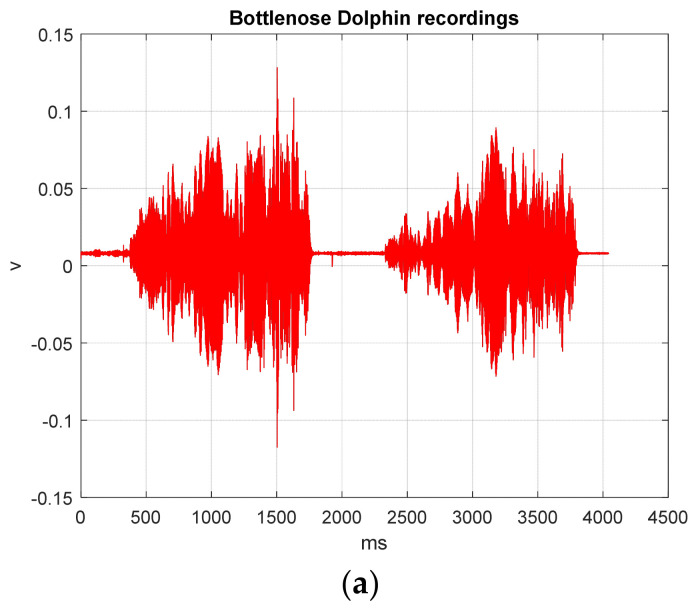

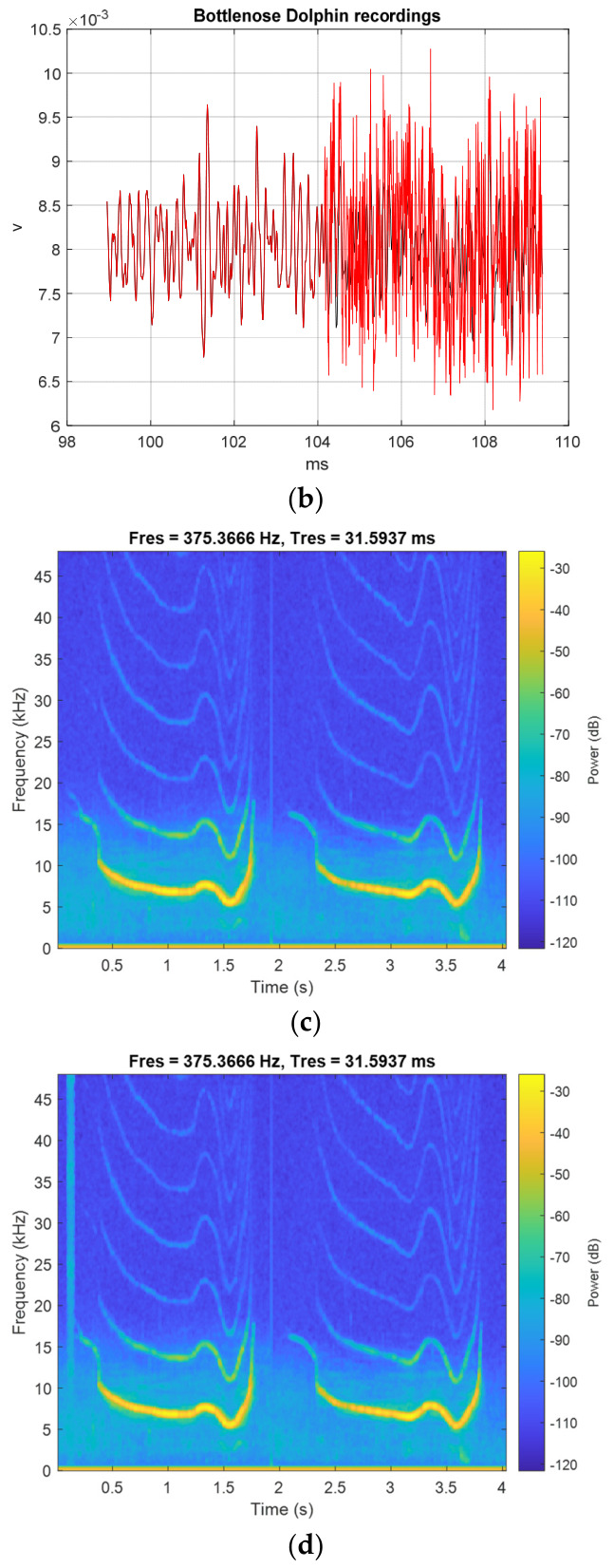
BDV (tbio_a_1613265_sm9612) processed with the MCHMTIEHM: (**a**) the time interval from 0 to 4043.6 ms; (**b**) the time interval from 98.96 to 109 ms; (**c**) the spectrogram of original BDV (tbio_a_1613265_sm9612); (**d**) the spectrogram of BDV (tbio_a_1613265_sm9612) hiding the encrypted signal. Black line, original BDV; red line, BDV hiding the encrypted signal.

For the TI01 MCHM encryption time series, the MCHM exhibits improved unpredictability. Notably, the red line conceals the black line. Figure 5a shows the BDV (tbio_a_1613265_sm9612) processed with the MCHMTIEHM in the time interval from 0 ms to 4043.6 ms. The TI01 MCHM encryption signal was embedded into the noise of the BDV (tbio_a_1613265_sm9612) in the time interval from 98.96 to 109 ms (Figure 5b). Figure 5c shows the spectrogram of the original BDV (tbio_a_1613265_sm9612) in the time interval 0 to 4043.6 ms. Figure 5d shows the spectrogram of BDV (tbio_a_1613265_sm9612) hiding the encrypted signal in the time interval 0 to 4043.6 ms. Figure 5c,d are approximately the same, and the proposed MCHMTIEHM yields superior hiding performance in the spectrogram domain.

**Figure 6:**
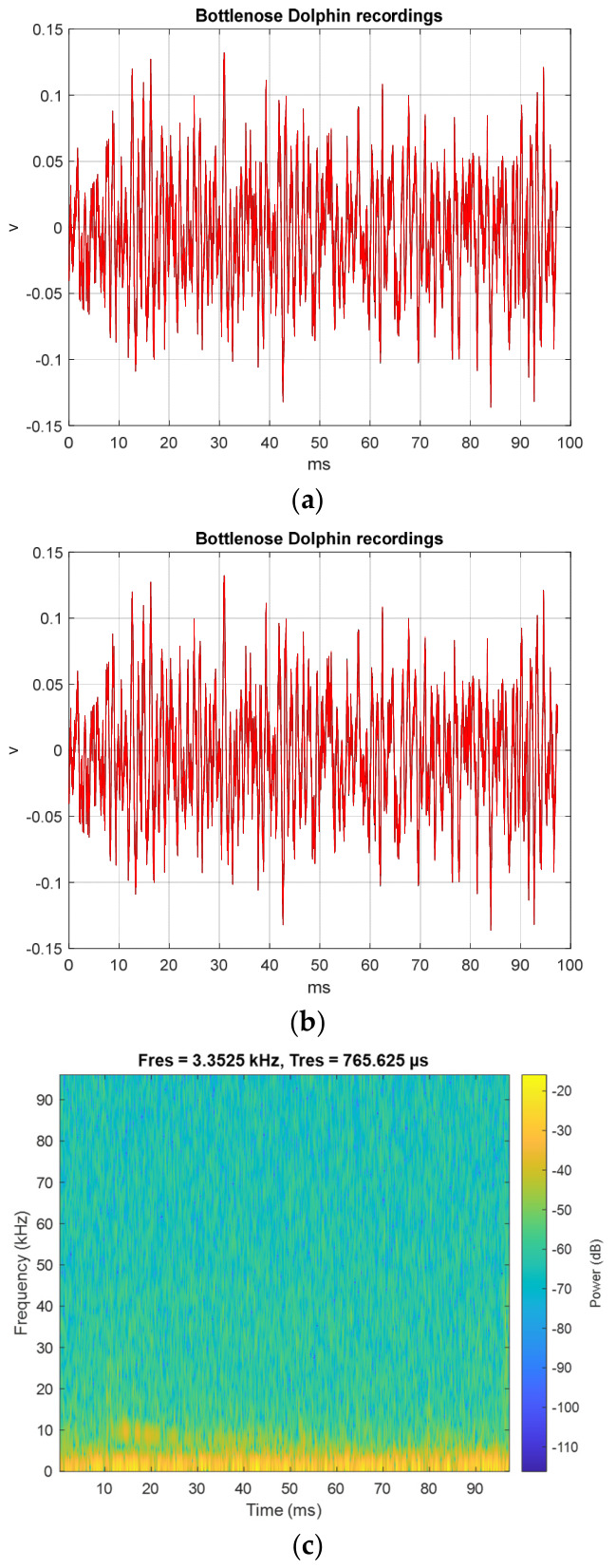

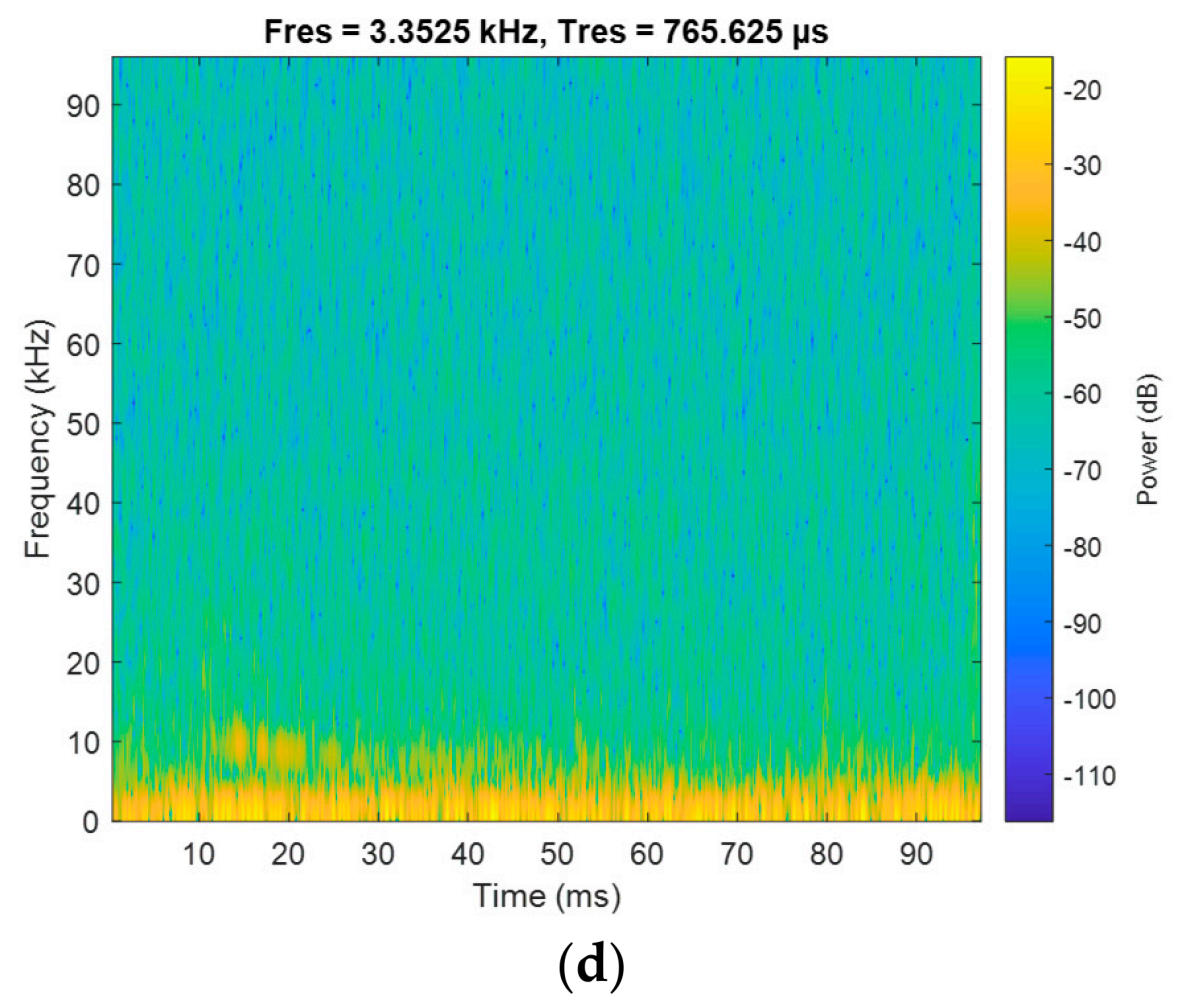
BDV (006_whistle) processed with the MCHMTIEHM: (**a**) the time interval from 0 to 97.30 ms; (**b**) the time interval from 49.48 to 54.80 ms; (**c**) the spectrogram of original BDV (006_whistle); (**d**) the spectrogram of BDV (006_whistle) hiding the encrypted signal. Black line, original BDV; red line, BDV hiding the encrypted signal.

Figure 6a shows the BDV (006_whistle) processed with MCHMTIEHM in the time interval from 0 to 97.30 ms. The TI01 MCHM encryption signal was embedded in the shape of the BDV (006_whistle) in the time interval from 49.48 to 54.80 ms (Figure 6b). Figure 6c shows the spectrogram of the original BDV (006_whistle) in the time interval 0 to 97.30 ms. Figure 6d shows the spectrogram of BDV (006_whistle) hiding the encrypted signal in the time interval 0 to 97.30 ms. Figure 6c,d are approximately the same, and the proposed MCHMTIEHM yields superior hiding performance in the spectrogram domain.

From Figure 4, Figure 5 and Figure 6, it can be seen that the proposed MCHMTIEHM achieves excellent concealment in BDVs, with the TI01 MCHM-encrypted and hiding signals being unrecognizable to the human eye.

**Figure 7:**
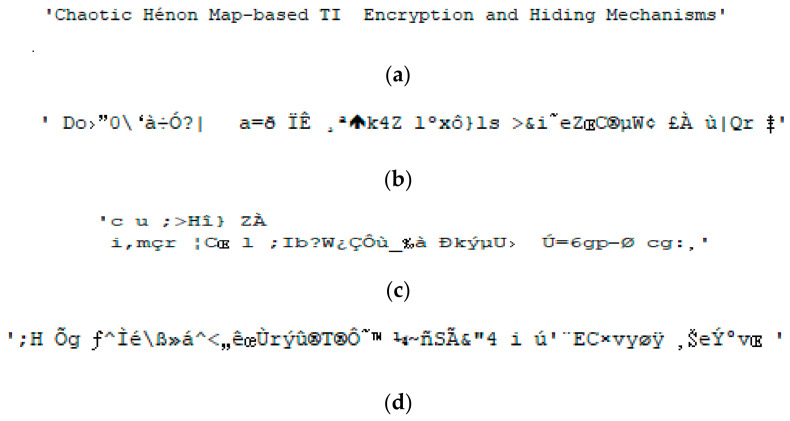
Decryption outcomes for TI01: (**a**) Correct de-hiding and correct TI decryption, ${SP}_{x}=0.63$, $n_{I}=10,000$; (**b**) correct de-hiding and error TI decryption, ${SP}_{x}=0.631$, $n_{I}=9700$; (**c**) correct de-hiding and error TI decryption, ${SP}_{x}=0.632$, $n_{I}=9800$; and (**d**) correct de-hiding and error TI decryption, ${SP}_{x}=0.633$, $n_{I}=9900$.

**Figure 8:**
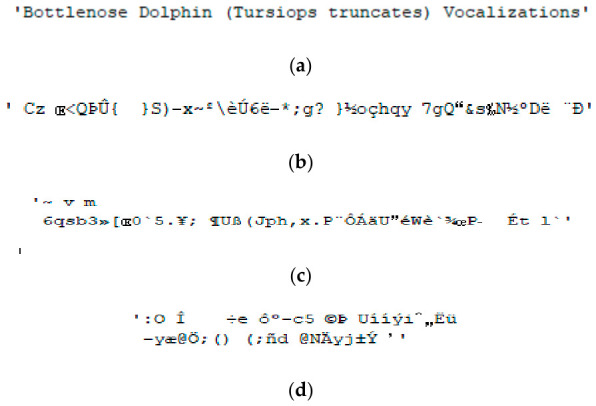
Decryption outcomes for TI02: (**a**) Correct de-hiding and correct TI decryption, ${SP}_{x}=0.63$, $n_{I}=10,000$; (**b**) correct de-hiding and error TI decryption, ${SP}_{x}=0.631$, $n_{I}=9700$; (**c**) correct de-hiding and error TI decryption, ${SP}_{x}=0.632$, $n_{I}=9800$; (**d**) correct de-hiding and error TI decryption, ${SP}_{x}=0.633$, $n_{I}=9900$.

**Figure 9:**
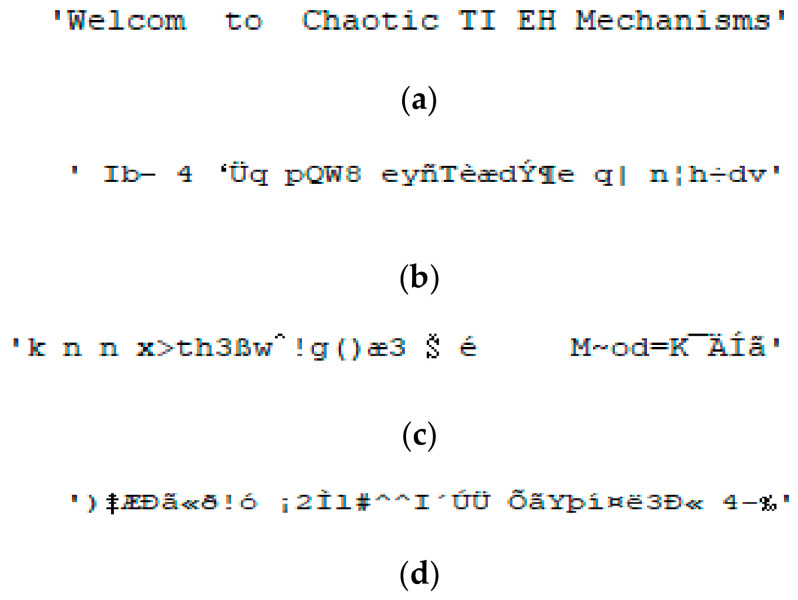
Decryption outcomes for TI03: (**a**) Correct de-hiding and correct TI decryption, ${SP}_{x}=0.63$, $n_{I}=10,000$; (**b**) correct de-hiding and error TI decryption, ${SP}_{x}=0.631$, $n_{I}=9700$; (**c**) correct de-hiding and error TI decryption, ${SP}_{x}=0.632$, $n_{I}=9800$; (**d**) correct de-hiding and error TI decryption, ${SP}_{x}=0.633$, $n_{I}=9900$.

Figure 7a shows TI01 after correct de-hiding and correct TI decryption, while Figure 7b–d show TI01 with correct de-hiding and erroneous TI decryptions. TI02 and TI03 are ‘Bottlenose Dolphin (*Tursiops truncatus*) Vocalizations’ and ‘Welcome to Chaotic TI EH Mechanisms’, respectively. Figure 8a and Figure 9a show TI02 and TI03 with correct de-hiding and correct TI decryption, respectively. Figure 8b–d show TI02 with correct de-hiding and erroneous TI decryptions. Figure 9b–d show TI03 with correct de-hiding and erroneous TI decryptions.

The MCHM decryption parameters ${\alpha ,\;\beta ,\;SP}_{x},\;{SP}_{y},\;\delta_{SL},\;{and\;n}_{I}$ were set to 1.4, 0.3, 0.63, 0.19, 0.1, and 10,000, respectively, to obtain the results shown in Figure 7, Figure 8 and Figure 9, while the MCHM encryption parameters were $\alpha ,\beta ,{SP}_{y},\;{and\;\delta }_{SL}$ are 1.4, 0.3, 0.19, and 0.1, respectively, in the same figures. In particular, the MCHM encryption and decryption parameters $\alpha ,\beta ,{SP}_{y},\;{and\;\delta }_{SL}$ were identical.

The MCHM encryption parameters ${SP}_{x}\;and\;n_{I}$ were set as 0.63 and 10,000, respectively, to obtain the outcomes shown in Figure 7a, Figure 8a and Figure 9a, with correct de-hiding and correct decryption achieved for TI01, TI02, and TI03. The test indicated superior correct de-hiding and decryption results, and when the correct encryption and decryption parameters were used, the TI was completely recovered and readable.

The MCHM encryption parameters ${SP}_{x}\;and\;n_{I}$ were set as 0.631 and 9700, respectively, for Figure 7b, Figure 8b and Figure 9b; 0.632 and 9800, respectively, for Figure 7c, Figure 8c and Figure 9c; and 0.633 and 9900, respectively, for Figure 7d, Figure 8d and Figure 9d. As the MCHM encryption and decryption parameters ${SP}_{x}\;and\;n_{I}$ were not identical, TI01, TI02, and TI03 showed correct de-hiding performance but erroneous decryption ability. The test indicated superior correct de-hiding and error decryption results, as the TI was unrecoverable and unrecognizable under these parameter settings.

## 4. Average Modified ACCs and Modified UACIs for TI Hiding Performance Evaluation

The modified ACC and modified UACI metrics were calculated to evaluate the hiding performance for MCHM-encrypted TI using BDVs.

The ACC and UACI are the two most common metrics used to evaluate the strength of image 2D encryption ciphers [30]. The modified ACC is calculated for the one-dimensional (1D) BDVs as follows:

(8) $$\mathrm{modified}\mathrm{ACC}=\frac{\sum_{i=1}^{N}\left(A_{l}-\alpha_{a})(B_{l}-\alpha_{b}\right)}{\sigma_{a}\sigma_{b}}$$

where $A_{i},\;\alpha_{a}$, $\sigma_{a},$ and *N* are the *i*th average, standard deviation, amplitude, and length of the original BDV, and $B_{i},\;\alpha_{b}$, and $\sigma_{b}$ are the *i*th average, standard deviation, and amplitude of the BDV, including the MCHM TI EH signal, respectively. When the modified ACC value is 1, the original BDV and the BDV with the MCHM TI EH signal are identical.

The modified UACI is calculated for the 1D BDVs as follows:

(9) $$\mathrm{modified}\mathrm{UACI}=\frac{1}{N}\frac{\left|\sum_{i=1}^{N}A_{i}-B_{i}\right|}{S_{max}}$$

where $A_{i}\;{and\;S}_{a}$ are the *i*th amplitude and maximum amplitude of the original BDV, respectively, and $B_{i}\;$ and $S_{b}$ are the *i*th amplitude and maximum amplitude of the BDV with the MCHM TI EH signal, respectively.

(10) $$S_{max}=S_{a}\;\;\;\;\;\;\;\;\left|S_{a}\right|\geq \left|S_{b}\right|$$

$$S_{max}=S_{b}\;\;\;\;\;\;\;\;\left|S_{a}\right|<\left|S_{b}\right|$$

**Figure 10:**
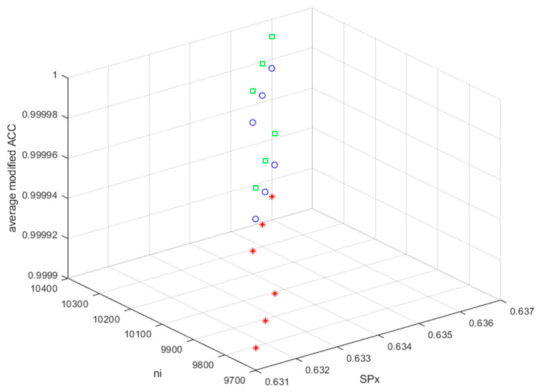
The average modified ACC values for MCHM TI (TI01, TI02, and TI03) hiding (blue o: BDV, tbio_a_1613265_sm9609; green □: BDV, tbio_a_1613265_sm9612; red *: BDV, 006_whistle).

**Figure 11:**
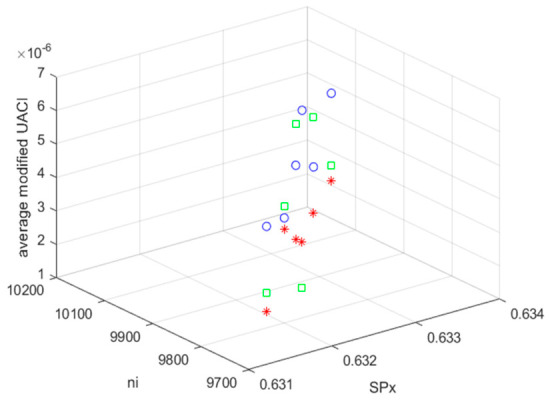
The average modified UACI values for MCHM TI (TI01, TI02, and TI03) hiding (blue o: BDV, tbio_a_1613265_sm9609; green □: BDV, tbio_a_1613265_sm9612; red *: BDV, 006_whistle).

When the values of the modified UACI and *ACC* are 0 and 1, respectively, the original BDV and the BDV with the MCHM TI EH signal are identical. Figure 10 and Figure 11 show the average modified ACC and average modified UACI values for hiding TI (TI01, TI02, and TI03).

The MCHM decryption parameters ${\alpha ,\;\beta ,\;SP}_{x},\;{SP}_{y},\;\delta_{SL},\;{and\;n}_{I}$ were set as 1.4, 0.3, 0.63, 0.19, 0.1, and 10,000, respectively, and the MCHM encryption parameters $\alpha ,\beta ,{SP}_{y},\;{and\;\delta }_{SL}$ were set as 1.4, 0.3, 0.19, and 0.1, respectively, to obtain the values shown in Figure 10 and Figure 11. Thus, the MCHM encryption and decryption parameters $\alpha ,\beta ,{SP}_{y},\;{and\;\delta }_{SL}$ were identical.

The average modified ACCs (TI01, TI02, and TI03) were 0.99997549, 0.99997551, 0.99997550, 0.99997552, 0.99997550, and 0.99997554 for the MCHM encryption parameters (${SP}_{x},\;n_{I})$ = (0.631, 9700), (0.632, 9800), (0.633, 9800), (0.634, 10,100), (0.635, 10,200), and (0.636, 10,300), respectively. The MCHM TI EH signal was embedded in the noise of BDV (tbio_a_1613265_sm9609), and the average modified ACC value was 0.99997551.

The average modified ACCs (TI01, TI02, and TI03) were 0.99999117, 0.99999120, 0.99999119, 0.99999121, 0.99999118, and 0.99999124 for the MCHM encryption parameters (${SP}_{x},\;n_{I})$ = (0.631, 9700), (0.632, 9800), (0.633, 9800), (0.634, 10,100), (0.635, 10,200), and (0.636, 10,300), respectively. The MCHM TI EH signal was embedded in the BDV noise (tbio_a_1613265_sm9612), and the average modified ACC value was 0.99999120.

The average modified ACCs (TI01, TI02, and TI03) were 0.99991116, 0.99991132, 0.99991126, 0.99991138, 0.99991123, and 0.99991155 for the MCHM encryption parameters (${SP}_{x},\;n_{I})$ = (0.631, 9700), (0.632, 9800), (0.633, 9800), (0.634, 10,100), (0.635, 10,200), and (0.636, 10,300), respectively. The MCHM TI EH signal was embedded in the contour of the BDV (006_whistle), and the average modified ACC value was 0.99991132.

The average modified ACC values for all BDVs (tbio_a_1613265_sm9609, tbio_a_1613265_sm9612, and 006_whistle) were very close to 1, and the hiding performances for BDV (tbio_a_1613265_sm9609) and BDV (tbio_a_1613265_sm9612) were better than that for BDV (006_whistle). The hiding performance of the MCHM TI EH signals embedded in BDV noise was better than that of those embedded in BDV contours. The MCHMTIEHM showed excellent concealment performance (i.e., LPD).

The average modified UACIs (TI01, TI02, and TI03) were $4.59\times 10^{-6},$ $4.15\times 10^{-6},$ $6.66\times 10^{-6},$ $2.19\times 10^{-6},$ $2.23\times 10^{-6},$ and $4.74\times 10^{-6}$ for the MCHM encryption parameters (${SP}_{x},\;n_{I})$ = (0.631, 9700), (0.632, 9800), (0.633, 9800), (0.634, 10,100), (0.635, 10,200), and (0.636, 10,300), respectively. The MCHM TI EH signal was embedded in the noise of BDV (tbio_a_1613265_sm9609), and the average modified UACI value was $4.09\times 10^{-6}$.

The average modified UACIs (TI01, TI02, and TI03) were $1.02\times 10^{-6}$, $9.19\times 10^{-6},$ $1.36\times 10^{-6}$, $4.85\times 10^{-6}$, $7.07\times 10^{-6}$, and $1.05\times 10^{-6}$ for the MCHM encryption parameters (${SP}_{x},n_{I})$ = (0.631, 9700), (0.632, 9800), (0.633, 9800), (0.634, 10,100), (0.635, 10,200), and (0.636, 10,300), respectively. The MCHM TI EH signal was embedded in the noise of BDV (tbio_a_1613265_sm9612), and the average modified UACI value was $4.09\times 10^{-6}$.

The average modified UACIs (TI01, TI02, and TI03) were $2.05\times 10^{-6}$, $1.85\times 10^{-6}$, $2.74\times 10^{-6}$, $9.78\times 10^{-6}$, $1.42\times 10^{-6}$, and $2.12\times 10^{-6}$ for the MCHM encryption parameters (${SP}_{x},n_{I})$ = (0.631, 9700), (0.632, 9800), (0.633, 9800), (0.634, 10,100), (0.635, 10,200), and (0.636, 10,300), respectively. The MCHM TI EH signal was embedded in the contour of BDV (006_whistle), and the average modified UACI value was $3.33\times 10^{-6}$.

The average modified UACI values for all BDVs (tbio_a_1613265_sm9609, tbio_a_1613265_sm9612, and 006_whistle) were very close to 0. The hiding performances for BDV (tbio_a_1613265_sm9609) and BDV (tbio_a_1613265_sm9612) were better than that for BDV (006_whistle). The hiding performance for the MCHM TI EH signals embedded in the noise of the BDV was better than that for the signals embedded in their contours. Again, the MCHMTIEHM showed excellent undetectable (LPD) performance.

## 5. Encryption Performance in Terms of the Average NCSSBRs, NCBRs, and NCCRs for Correct and Erroneous TI Decryption

The encryption performance of the original and decrypted TI was evaluated using NCSSBRs, NCBRs, and NCCRs. The NCBR metric for encryption is defined as follows [30]:

(11) $$NCBR=\frac{\sum_{j=1}^{M}D(j)}{M}\times 100\%$$

$$D\left(j\right)=0\;C_{1}\left(j\right)=C_{2}\left(j\right)$$

$$D\left(j\right)=1\;C_{1}(j)\neq C_{2}(j)$$

where $C_{1}\left(j\right)$ and $C_{2}\left(j\right)$ are the *j*th bits of the original and correctly/erroneously decrypted ASCII TI bit streams, respectively, and *M* is the length of the original ASCII TI bit stream.

The NCSSBR metric for encryption is defined as follows:

(12) $$NCSSBR=\frac{\sum_{j=1}^{O}DSS(j)}{O}\times 100\%$$

$$DSS\left(j\right)=0\;{CSS}_{1}\left(j\right)={CSS}_{2}\left(j\right)$$

$$DSS\left(j\right)=1\;{CSS}_{1}(j)\neq {CSS}_{2}(j)$$

where ${CSS}_{1}\left(j\right)$ and ${CSS}_{2}\left(j\right)$ are the *j*th bits of the original and correctly/erroneously decrypted ASCII SS bit streams, respectively, and *O* is the length of the original ASCII TI SS bit stream (*O* is given by M×SF).

The NCCR metric for encryption is defined as follows:

(13) $$NCCR=\frac{\sum_{j=1}^{P}DC(j)}{P}\times 100\%$$

$$DC\left(j\right)=0\;{CC}_{1}\left(j\right)={CC}_{2}\left(j\right)$$

$$DC\left(j\right)=1\;{CC}_{1}(j)\neq {CC}_{2}(j)$$

where ${CC}_{1}\left(j\right)$ and ${CC}_{2}\left(j\right)$ are the *j*th characters of the original and correctly/erroneously decrypted TI characters, respectively, and *P* is the number of original TI characters.

**Figure 12:**
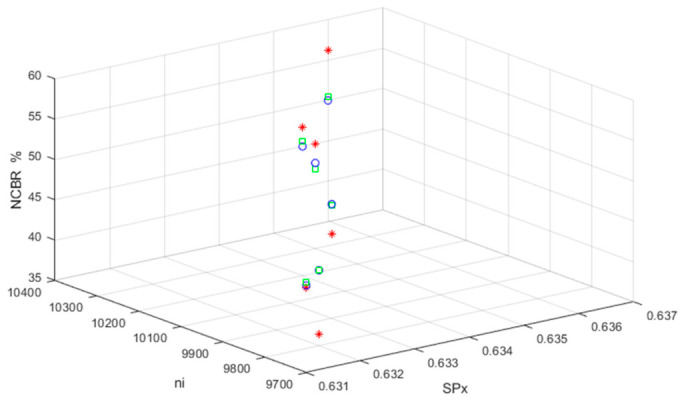
The NCBRs for MCHM TI with correct de-hiding and error decryptions (blue o: TI01; green □: TI02; red *: TI03).

**Figure 13:**
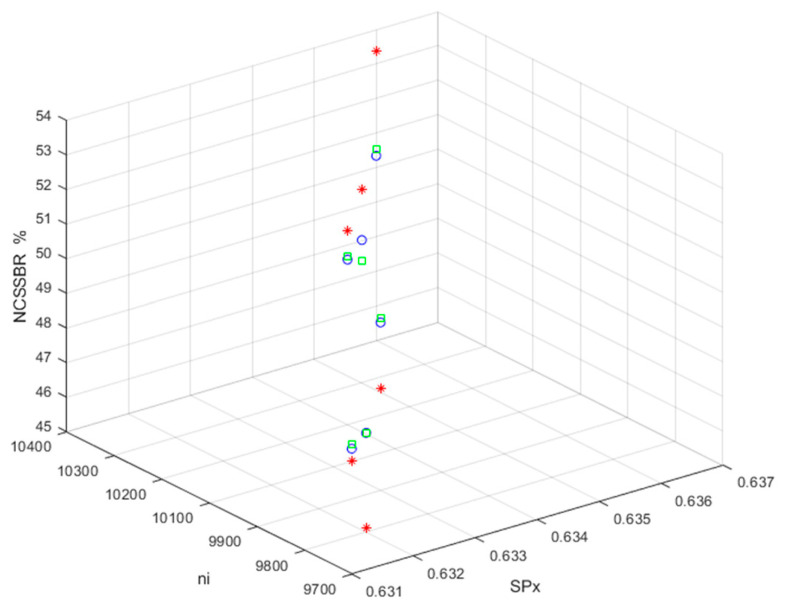
The *NCSSBRs* for MCHM TIs with correct de-hiding and error decryptions (blue o: TI01; green □: TI02; red *: TI03).

**Figure 14:**
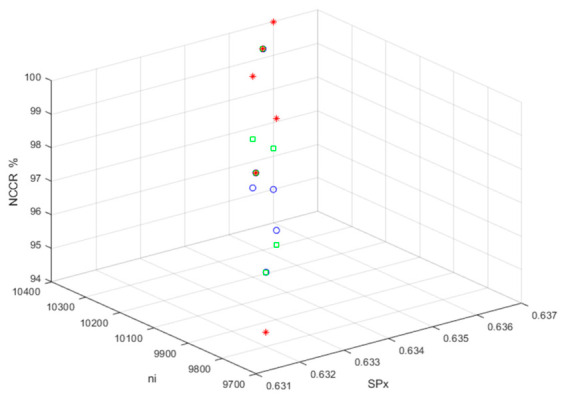
The NCCRs for MCHM TI with correct de-hiding and error decryptions (blue o: TI01; green □: TI02; red *: TI03).

The obtained NCBR, NCSSB, and NCCR values were 50%, 50%, and 100%, respectively, indicating that the original TI (TI01, TI02, and TI03) and the MCHM TI with correct de-hiding and error decryptions are optimal. Figure 12, Figure 13 and Figure 14 show the performance in terms of the NCBRs, NCSSBRs, and NCCRs, respectively, for MCHM TI with correct de-hiding and error decryptions.

The MCHM decryption parameters ${\alpha ,\beta ,SP}_{x},{SP}_{y},\delta_{SL},\;{and\;n}_{I}$ were set as 1.4, 0.3, 0.63, 0.19, 0.1, and 10,000, respectively, while the MCHM encryption parameters $\alpha ,\beta ,{SP}_{y},\;and\delta_{SL}$ were set as 1.4, 0.3, 0.19, and 0.1, respectively, to obtain the values shown in Figure 12, Figure 13 and Figure 14. Thus, the MCHM encryption and decryption parameters $\alpha ,\beta ,{SP}_{y},\;and\delta_{SL}$ were identical. The NCBRs of TI01 were 45.83%, 44.34%, 49.17%, 51.04%, 45.63%, and 50.00% for the MCHM encryption parameters (${\;SP}_{x},\;n_{I})$ = (0.631, 9700), (0.632, 9800), (0.633, 9800), (0.634, 10,100), (0.635, 10,200), and (0.636, 10,300), respectively, in the scenario of correct de-hiding from BDV (tbio_a_1613265_sm9609).

The MCHM encryption parameters were the same for TI02 and TI03. The average NCBR for TI01 was 47.67%, and the NCBRs for TI01 with correct de-hiding from BDV (tbio_a_1613265_sm9609) were the same as those with correct de-hiding from BDV (tbio_a_1613265_sm9612) and BDV (006_whistle). The NCBRs of TI01 did not correlate with the embedded BDVs. The NCBRs of TI02 were 46.23%, 44.34%, 49.06%, 51.65%, 44.81%, and 50.47% when embedded in BDV (tbio_a_1613265_sm9609), and the average NCBR of TI02 was 47.78%. The NCBRs of TI03 were 45.49%, 36.46%, 45.49%, 53.47%, 47.92%, and 56.25% when embedded in BDV (tbio_a_1613265_sm9609), and the average NCBR of TI03 was 47.51%. The NCBRs of TI01, TI02, and TI03 were close to 50%, indicating that MCHM encryption yields excellent and robust performance for correct de-hiding and error decryption.

The NCSSBRs of TI01 were 48.63%, 47.88%, 49.86%, 49.77%, 49.12%, and 50.35% for the MCHM encryption parameters (${SP}_{x},n_{I})$ = (0.631, 9700), (0.632, 9800), (0.633, 9800), (0.634, 10,100), (0.635, 10,200), and (0.636, 10,300), respectively, with correct de-hiding from BDV (tbio_a_1613265_sm9609). The MCHM encryption parameters were the same for TI02 and TI03. The average NCSSBR of TI01 was 49.27%, and the NCBRs for TI01 with correct de-hiding from BDV (tbio_a_1613265_sm9609) were the same as those for TI01 with correct de-hiding from BDV (tbio_a_1613265_sm9612) and BDV (006_whistle). The NCBRs of TI01 did not correlate with the embedded BDVs. The NCSSBRs of TI02 were 48.77%, 47.88%, 50.00%, 49.89%, 48.51%, and 50.55% with correct de-hiding from BDV (tbio_a_1613265_sm9609), and the average NCBR of TI02 was 49.27%. The NCBRs of TI03 were 48.26%, 45.14%, 47.96%, 50.62%, 50.58%, and 53.40% with correct de-hiding from BDV (tbio_a_1613265_sm9609), and the average NCBR of TI03 was 49.33%. The NCSSBRs of TI01, TI02, and TI03 were all close to 50%. Thus, the MCHM encryption showed excellent and robust performance for correct de-hiding and error decryption.

The NCCRs of TI01 were 100.00%, 96.23%, 96.67%, 96.67%, 100.00%, and 95.00% for the MCHM encryption parameters (${SP}_{x},n_{I})=$ (0.631, 9700), (0.632, 9800), (0.633, 9800), (0.634, 10,100), (0.635, 10,200), and (0.636, 10,300), respectively, with correct de-hiding from BDV (tbio_a_1613265_sm9609). The MCHM encryption parameters were the same for TI02 and TI03. The average NCCR of TI01 was 97.43%. The NCCRs of TI01 with correct de-hiding from BDV (tbio_a_1613265_sm9609) were the same as those of TI01 with correct de-hiding from BDV (tbio_a_1613265_sm9612) and BDV (006_whistle). The NCCRs of TI01 did not correlate with the embedded BDVs. The NCCRs of TI02 were 100.00%, 96.23%, 96.23%, 98.11%, 100.00%, and 96.23% with correct de-hiding from BDV (tbio_a_1613265_sm9609), and the average NCCR of TI02 was 97.80%. The NCCRs of TI03 were 100.00%, 94.44%, 100.00%, 100.00%, 100.00%, and 100.00% with correct de-hiding from BDV (tbio_a_1613265_sm9609), and the average NCBR of TI03 was 99.07%. The NCSSBRs of TI01, TI02, and TI03 were close to 50%. Thus, the MCHM encryption had excellent and robust performance for correct de-hiding and error decryption. There were 60, 53, and 36 characters in TI01, TI02, and TI03, respectively. The average NCCRs of TI01, TI02, and TI03 with correct de-hiding and error decryption were 97.43%, 97.80%, and 99.07%, respectively. As such, when the number of TI characters decreased, the NCCR values for TI with correct de-hiding and error-decryption were improved.

## 6. Discussion

The performance of the proposed MCHMTIEHM was explored using three BDVs and three TI examples. Table 1 details the performance of the proposed MCHMTIEHM in the scenario of correct de-hiding and correct decryption.

Figure 4, Figure 5 and Figure 6 above show that the proposed MCHMTIEHM achieved excellent concealment of the BDVs, and the TI01 MCHMEH signals were unrecognizable to the human eye. Figure 7a, Figure 8a and Figure 9a show that the correctly unveiled and correctly decrypted pieces of TI were completely recovered and recognizable. As the modified ACC was 1 and the modified UACI was 0, the original BDV and the BDV with the MCHM TI EH signals were identical. The average modified ACC values (TI01, TI02, and TI03) were 0.99997547, 0.99999116, and 0.99991116 for tbio_a_1613265_sm9609, tbio_a_1613265_sm9612, and 006_whistle, respectively. Furthermore, the average modified UACI values (TI01, TI02, and TI03) were $3.96\times 10^{-6},\;3.57\times 10^{-6}$, and $4.00\times 10^{-6}$ for tbio_a_1613265_sm9609, tbio_a_1613265_sm9612, and 006_whistle, respectively. The original BDVs and the BDVs with the MCHM TI EH signals appear to be almost the same. The average NCBR, NCSSBR, and NCCR were 0%, 0%, and 0%, respectively, reflecting both correct de-hiding and correct decryption.

The average number of TI chars, average modified ACC, average modified UACI, average NCBR, NCSSBR, and NCCR were 49.67, 0.99995934, $3.84\times 10^{-6}$, 0%, 0$, and 0%, respectively, in Table 1. The proposed MCHMTIEHM has excellent and undetectable (i.e., LPD) performance with respect to TI hiding and yields correct de-hiding and decryption results.

Table 2 details the performance of the proposed MCHMTIEHM in the scenario of correct de-hiding and error decryption. The MCHM encryption and decryption parameters of $\alpha ,\;\beta ,\;{SP}_{y},\;and\;\delta_{SL}$ were the same, while those of ${\;SP}_{x}$ and $n_{I}\;differed\;for\;the\;cases\;shown\;in$ Figure 7b, Figure 8b and Figure 9b. In these cases, TI01, TI02, and TI03 yielded correct de-hiding and error decryption outcomes. For the test TI, the correct de-hiding and error decryption results were robust and superior, and they were not recoverable or recognizable.

With different encryption parameters ${SP}_{x}\;{and\;n}_{I},$ different MCHM encryption signals were generated. Thus, the average modified ACC and modified UACI values of the original BDVs (tbio_a_1613265_sm9609) and the BDVs (tbio_a_1613265_sm9609) with the MCHM TI EH signal also differed. The average modified ACC values (TI01, TI02, and TI03) were 0.99997551, 0.99999120, and 0.99991132 for TI embedded in the BDVs tbio_a_1613265_sm9609, tbio_a_1613265_sm9612, and 006_whistle, respectively, while the respective average modified UACI values (TI01, TI02, and TI03) were $4.09\times 10^{-6}$, $4.09\times 10^{-6}$, and 3.33 $\times 10^{-6}$. The average modified ACC and modified UACI values were 0.99995924 and $3.84\times 10^{-6}$, respectively, in Table 2. The proposed MCHMTIEHM thus showed excellent and undetectable (LPD) hiding performance.

As the NCBR, NCSSB, and NCCR values were 50%, 50%, and 100%, respectively, the original TI and the MCHM TI with the correct de-hiding and error decryptions exhibited optimal performance. The average NCBR, NCSSB, and NCCR values were 47.65%, 49.29%, and 98.10%, respectively, in Table 2. Thus, the proposed MCHMTIEHM is considered to have excellent and robust correct de-hiding and error-decryption performance.

We previously proposed an F-OFDM-based covert underwater acoustic communication system that uses chaotic Hénon text encryption and hiding mechanisms, implemented with bottlenose dolphin vocalizations [31,32]. In particular, the amplitudes of the BDVs with EH TI signals were quantized using a word length of 16 bits. The underwater acoustic signal-to-noise ratio (SNR) was 6.14 dB, and the transmission bit error rate was $9.78\times 10^{-4}$. The NCBR and NCCR of the original TI and correct encryption were both 0%. The ACC and modified UACI of the original BDVs and EH TI were 0.9817 and 0.0057, respectively.

When the MCHM encryption parameters ${\alpha ,\beta ,SP}_{x},{SP}_{y},\delta_{SL},\;and\;n_{I}$ were set to 1.4, 0.3, 0.631, 0.19, 0.11, and 9700, and the MCHM decryption parameters ${\alpha ,\beta ,SP}_{x},{SP}_{y},\delta_{SL},\;and\;n_{I}$ set to 1.4, 0.3, 0.63, 0.19, 0.1, and 10,000, respectively, the decryption was erroneous. The SNR was 6.14 dB, and the transmission bit error rate was $8.87\times 10^{-4}.$ The NCBR and NCCR of the original TI and error decryption were 46.67% and 98.33%, respectively, and the ACC and modified UACI between the original and EH TI BDVs were 0.9861 and 0.0051, respectively.

**Figure 15:**
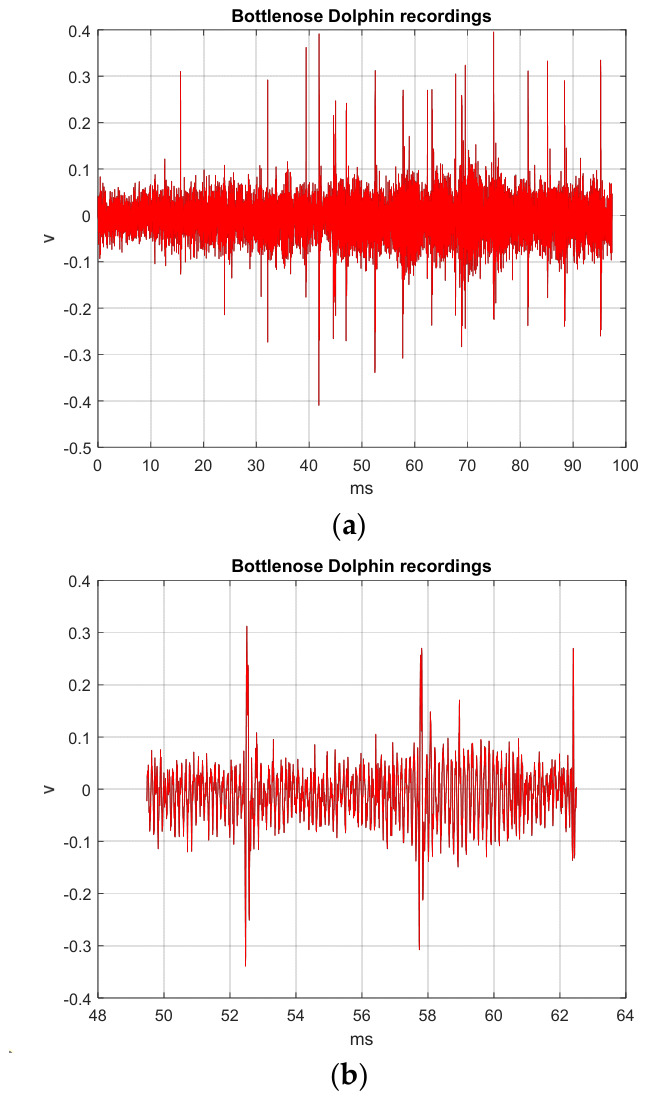

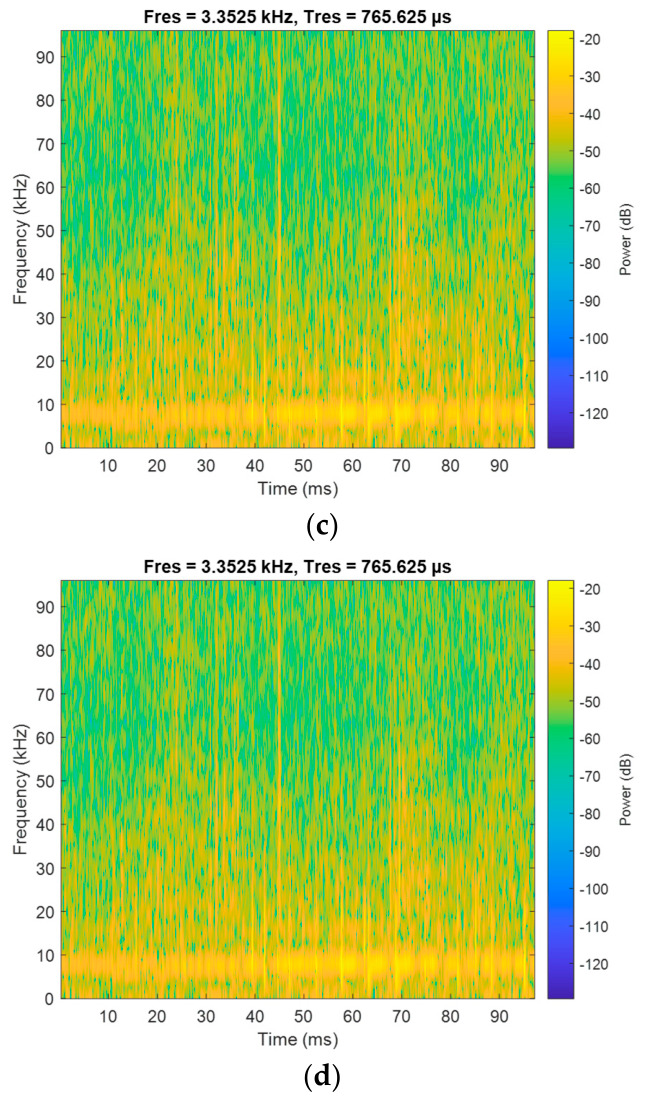
TBDWV (20230219_009_10_BottlenoseDolphin) processed with the MCHMTIEHM: (**a**) the time interval from 0 to 97.40 ms; (**b**) the time interval from 49.48 to 62.50 ms; (**c**) the spectrogram of original TBDWV (20230219_009_10_BottlenoseDolphin); (**d**) the spectrogram of TBDWV (20230219_009_10_BottlenoseDolphin) hiding the encrypted signal. Black line, original BDV; red line, BDV hiding the encrypted signal.

In addition, the Taiwan-bottlenose dolphin-whistle vocalization (TBDWV) (20230219_009_10_BottlenoseDolphin.wav, accessed on 04.08.2026) used in this study was obtained from the website of the Hualien Formosa Association, Hualien, Taiwan [33]. Figure 15a shows the TBDWV (20230219_009_10_BottlenoseDolphin) processed with MCHMTIEHM in the time interval from 0 to 97.40 ms. The ‘Welcome to Bottlenose Dolphin Vocalization Signal Processing (TI04)’ MCHM encryption signal was embedded in the shape of the TBDWV (20230219_009_10_BottlenoseDolphin) in the time interval from 49.48 to 62.50 ms (Figure 15b).

Figure 15c shows the spectrogram of the original TBDWV (20230219_009_10_BottlenoseDolphin) in the time interval 0 to 97.40 ms. Figure 15d shows the spectrogram of TBDWV (20230219_009_10_BottlenoseDolphin) hiding the encrypted signal in the time interval 0 to 97.40 ms. Figure 15c,d are approximately the same, and the proposed MCHMTIEHM yields superior hiding performance in the spectrogram domain.

The ${\alpha ,\beta ,SP}_{x},{SP}_{y},\delta_{SL},\;and\;n_{I}\;of$ the decryption parameters were 1.4, 0.3, 0.63, 0.19, 0.1, and 10,000, respectively, across all Table 3, Table 4, Table 5, Table 6, Table 7, Table 8 and Table 9.

Table 3 shows the correct de-hiding and decryption text, along with the NBCR, NCCR, ACC, and UACI values, using the encryption parameters (1.4, 0.3, 0.63, 0.19, 0.1, 10,000). The SNR was 6.14 dB, and the transmission bit error rate was $9.04\times 10^{-4}.$

The NCBR and NCCR of the original TI were 0% and 0%, respectively, and the ACC and modified UACI between the original and EH TI BDVs were 0.9886 and 0.0017, respectively.

The transmission BERs were $9.04\times 10^{-4}$ (Table 3), $9.57\times 10^{-4}$ (Table 4), $8.35\times 10^{-4}\;$, (Table 5), $9.91\times 10^{-4}\;$ (Table 6), $8.87\times 10^{-4}$ (Table 7), $\;9.91\times 10^{-4}$ (Table 8), and $8.91\times 10^{-4}\;$ (Table 9). The underwater voice transmission had a BER quality of service $10^{-3}$. The proposed an F-OFDM-based covert underwater acoustic communication system using chaotic Hénon text encryption and hiding mechanisms with a BER below $10^{-3}$.

The average modified ACCs were 0.9886 (Table 3), 0.9873 (Table 4), 0.9882 (Table 5), 0.9884  (Table 6), 0.9873 (Table 7), 0.9910  (Table 8), and 0.9875  (Table 9). The average modified UACIs were 0.0017 (Table 3), 0.0019 (Table 4), 0.0018  (Table 5), 0.0019  (Table 6), 0.0017 (Table 7), 0.0017 (Table 8), and 0.0018  (Table 9). For the hiding method, the optimal average modified ACC and UACI values were 1 and 0, respectively, and the proposed F-OFDM-based covert UAC scheme with an EH mechanism exhibited excellent average modified ACC and UACI performance in the hiding scenarios.

The average NCBR and NCCR were 0% (Table 3) for correct de-hiding and 0% (Table 3) for correct decryption text. The average NCBR and NCCR optimal values were 0% and 0%, respectively, for correct de-hiding and correct decryption, and the proposed EH mechanism has exhibited NCBR and NCCR performance in the correct de-hiding and correct decryption text scenarios.

For the correct de-hiding and error-decryption text scenarios, the average NCBR were 47.29% (Table 4), 51.04%  (Table 5), 48.33% (Table 6), 50.21% (Table 7), 45.42%  (Table 8), and 51.67% (Table 9).

For the correct de-hiding and error-decryption text scenarios, the average NCCR were 100% (Table 4), 98.83%  (Table 5), 100% (Table 6), 98.83% (Table 7), 98.33% (Table 8), and 98.33%  (Table 9).

The average NCBR and NCCR optimal values were 50% and 100%, respectively, for correct de-hiding and error decryption, and the proposed EH mechanism has exhibited NCBR and NCCR performance in the correct de-hiding and error decryption text scenarios.

From Table 4, Table 5, Table 6, Table 7, Table 8 and Table 9, the average SNR and BER were 6.14 dB $and\;9.25\times 10^{-4}$, respectively. The average modified ACC and modified UACI values were 0.9883 and 0.0018, respectively, in the hiding scenarios. The average NCBR and NCCR values were 48.99% and 99.22%, respectively, for the correct de-hiding and error-decryption text scenarios. Thus, the proposed F-OFDM-based covert UAC scheme showed excellent EH TI performance.

## 7. Conclusions

In this article, we present the MCHMTIEHM, which integrates the MCHM, SS, and BDVs. The hiding performance of four different BDVs was assessed in terms of the modified ACC and UACI metrics. Furthermore, the correct de-hiding and correct/erroneous decryptions of four TI pieces with character lengths of 60, 53, 36, and 60 were assessed using the NCBR, NCSSBR, and NCCR metrics. In terms of the hidden signals, correctly unveiled/correctly decrypted and correctly unveiled/erroneously decrypted TI scenarios were considered.

The test results indicated superior robustness and undetectable (LPD) TI EH performance. When the correct de-hiding and deciphering parameters were given as inputs, the test TI was completely recovered. However, input parameter errors yielded erroneous decryption of the test TI, which was unrecognizable to the human eye.

In the future, we plan to explore the following research topics in detail: filter-orthogonal frequency division multiplexing-based CUAC using chaotic logistic-sine and/or Hénon text encryption; SS and hiding mechanisms with BDVs; and large-capacity TI-hiding schemes utilizing chaotic SS strategies.

## Figures and Tables

**Table 1:** The performance of the proposed MCHMTIEHM in the correct de-hiding and correct decryption scenario.

BDV	TI	Number of TI Chars	TI Embedded Performance, Unrecognizable to the Human Eye	TI Completely Recovered	Modified ACC (TI01, TI02, and TI03)	Modified UACI	NCBR (%)	NCSSBR (%)	NCCR (%)
tbio_a_1613265_sm9609	01	60	Yes Figure 4a	Yes Figure 7a	0.99997547	$3.96\times 10^{-6}$ (average TI01, TI02, and TI03)	0	0	0
tbio_a_1613265_sm9609	02	53	Yes Figure 5a	Yes Figure 8a			0	0	0
tbio_a_1613265_sm9609	03	36	Yes Figure 6a	Yes Figure 9a			0	0	0
tbio_a_1613265_sm9612	01	60	Yes	Yes	0.9999116	$3.57\times 10^{-6}$ (average TI01, TI02, and TI03)	0	0	0
tbio_a_1613265_sm9612	02	53	Yes	Yes			0	0	0
tbio_a_1613265_sm9612	03	36	Yes	Yes			0	0	0
006_whistle	01	60	Yes	Yes	0.99991116	$4.00\times 10^{-6}$ (average TI01, TI02, and TI03)	0	0	0
006_whistle	02	53	Yes	Yes			0	0	0
006_whistle	03	36	Yes	Yes			0	0	0
average	-	49.67	Yes	Yes	0.99995926	$3.84\times 10^{-6}$	0	0	0

**Table 2:** The performance of the proposed MCHMTIEHM in the correct de-hiding and error decryption scenario.

BDV	TI	Number of TI Chars	Unrecoverable	Average Modified ACC (TI01, TI02, and TI03)	Average Modified UACI	Average NCBR (%)	Average NCSSBR (%)	Average NCCR (%)
tbio_a_1613265_sm9609	01	60	Yes Figure 7b–d	0.99997551	$4.09\times 10^{-6}$ (average TI01, TI02, and TI03)	47.67	49.27	97.43
tbio_a_1613265_sm9609	02	52	Yes Figure 8b–d			47.78	49.27	97.80
tbio_a_1613265_sm9609	03	36	Yes Figure 9b–d			47.51	49.33	99.07
tbio_a_1613265_sm9612	01	60	Yes	0.99999120	$4.09\times 10^{-6}$ (average TI01, TI02, and TI03)	47.67	49.27	97.43
tbio_a_1613265_sm9612	02	52	Yes			47.78	49.27	97.80
tbio_a_1613265_sm9612	03	36	Yes			47.51	49.33	99.07
006_whistle	01	60	Yes	0.99991132	$3.33\times 10^{-6}$ (average TI01, TI02, and TI03)	47.67	49.27	97.43
006_whistle	02	52	Yes			47.78	49.27	97.80
006_whistle	03	36	Yes			47.51	49.33	99.07
average	-	49.67	Yes	0.99995924	$3.84\times 10^{-6}$	47.65	49.29	98.10

**Table 3:** The performance of the proposed MCHMTIEHM in the correct de-hiding and correct decryption scenario (SNR = 6.14 dB, BER = 9.04 × 10^−4^).

Encryption Parameters $\left({\alpha ,\beta ,SP}_{x},{SP}_{y},\delta_{SL},n_{I}\right)$	Decryption Parameters $\left({\alpha ,\beta ,SP}_{x},{SP}_{y},\delta_{SL},n_{I}\right)$	Average *Modified* *ACC*	Average *Modified* *UACI*	Average *NC* *BR* (%)	Average *NC* *CR* (%)
(1.4, 0.3, 0.63, 0.19, 0.1, 10,000)	(1.4, 0.3, 0.63, 0.19, 0.1, 10,000)	0.9886	0.0017	0	0
Original text					
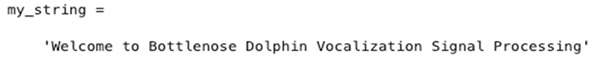					
Correct de-hiding and decryption text					
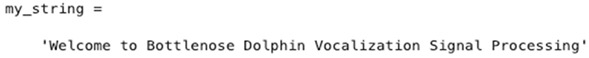					

**Table 4:** The performance of the proposed MCHMTIEHM in the correct de-hiding and error decryption scenario (SNR = 6.14 dB, BER = 9.57 × 10^−4^).

Encryption Parameters $\left({\alpha ,\beta ,SP}_{x},{SP}_{y},\delta_{SL},n_{I}\right)$	Decryption Parameters $\left({\alpha ,\beta ,SP}_{x},{SP}_{y},\delta_{SL},n_{I}\right)$	Average *Modified* *ACC*	Average *Modified* *UA* *CI*	Average *NC* *BR* (%)	Average *NC* *CR* (%)
(1.4, 0.3, 0.631, 0.19, 0.11, 9700)	(1.4, 0.3, 0.63, 0.19, 0.1, 10,000)	0.9873	0.0019	47.29	100
Original text					
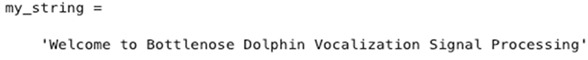					
Correct de-hiding and error decryption text					
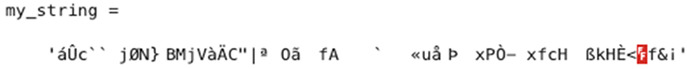					

**Table 5:** The performance of the proposed MCHMTIEHM in the correct de-hiding and error decryption scenario (SNR = 6.14 dB, BER = 8.35 × 10^−4^).

Encryption Parameters $\left({\alpha ,\beta ,SP}_{x},{SP}_{y},\delta_{SL},n_{I}\right)$	Decryption Parameters $\left({\alpha ,\beta ,SP}_{x},{SP}_{y},\delta_{SL},n_{I}\right)$	Average *Modified* *ACC*	Average *Modified* *UA* *CI*	Average *NC* *BR* (%)	Average *NC* *CR* (%)
(1.4, 0.3, 0.632, 0.19, 0.12, 9800)	(1.4, 0.3, 0.63, 0.19, 0.1, 10,000)	0.9882	0.0018	51.04	98.33
Original text					
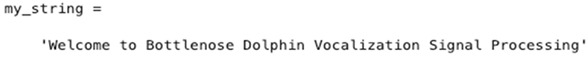					
Correct de-hiding and error decryption text					
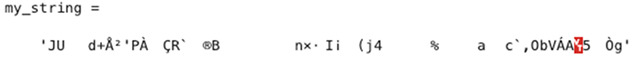					

**Table 6:** The performance of the proposed MCHMTIEHM in the correct de-hiding and error decryption scenario (SNR = 6.14 dB, BER = 9.92 × 10^−4^).

Encryption Parameters $\left({\alpha ,\beta ,SP}_{x},{SP}_{y},\delta_{SL},n_{I}\right)$	Decryption Parameters $\left({\alpha ,\beta ,SP}_{x},{SP}_{y},\delta_{SL},n_{I}\right)$	Average *Modified* *ACC*	Average *Modified* *UA* *CI*	Average *NC* *BR* (%)	Average *NC* *CR* (%)
(1.4, 0.3, 0.633, 0.19, 0.13, 9900)	(1.4, 0.3, 0.63, 0.19, 0.1, 10,000)	0.9884	0.0019	48.33	100
Original text					
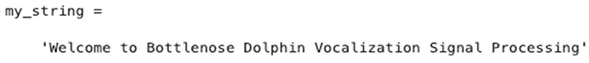					
Correct de-hiding and error decryption text					
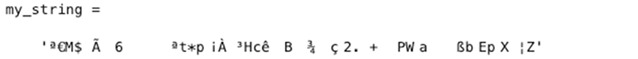					

**Table 7:** The performance of the proposed MCHMTIEHM in the correct de-hiding and error decryption scenario (SNR = 6.14 dB, BER = 8.87 × 10^−4^).

Encryption Parameters $\left({\alpha ,\beta ,SP}_{x},{SP}_{y},\delta_{SL},n_{I}\right)$	Decryption Parameters $\left({\alpha ,\beta ,SP}_{x},{SP}_{y},\delta_{SL},n_{I}\right)$	Average *Modified* *ACC*	Average *Modified* *UA* *CI*	Average *NC* *BR* (%)	Average *NC* *CR* (%)
(1.4, 0.3, 0.634, 0.19, 0.14, 10,100)	(1.4, 0.3, 0.63, 0.19, 0.1, 10,000)	0.9873	0.0017	50.21	98.33
Original text					
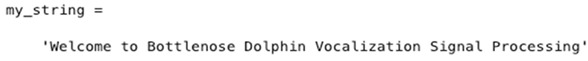					
Correct de-hiding and error decryption text					
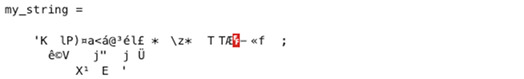					

**Table 8:** The performance of the proposed MCHMTIEHM in the correct de-hiding and error decryption scenario (SNR = 6.14 dB, BER = 9.91 × 10^−4^).

Encryption Parameters $\left({\alpha ,\beta ,SP}_{x},{SP}_{y},\delta_{SL},n_{I}\right)$	Decryption Parameters $\left({\alpha ,\beta ,SP}_{x},{SP}_{y},\delta_{SL},n_{I}\right)$	Average *Modified* *ACC*	Average *Modified* *UA* *CI*	Average *NC* *BR* (%)	Average *NC* *CR* (%)
(1.4, 0.3, 0.635, 0.19, 0.15, 10,200)	(1.4, 0.3, 0.63, 0.19, 0.1, 10,000)	0.9910	0.0017	45.42	98.33
Original text					
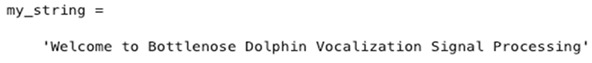					
Correct de-hiding and error decryption text					
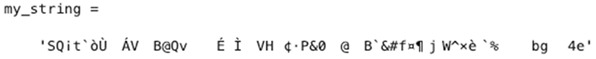					

**Table 9:** The performance of the proposed MCHMTIEHM in the correct de-hiding and error decryption scenario (SNR = 6.14 dB, BER = 8.91 × 10^−4^).

Encryption Parameters $\left({\alpha ,\beta ,SP}_{x},{SP}_{y},\delta_{SL},n_{I}\right)$	Decryption Parameters $\left({\alpha ,\beta ,SP}_{x},{SP}_{y},\delta_{SL},n_{I}\right)$	Average *Modified* *ACC*	Average *Modified* *UA* *CI*	Average *NC* *BR* (%)	Average *NC* *CR* (%)
(1.4, 0.3, 0.636, 0.19, 0.16, 10,300)	(1.4, 0.3, 0.63, 0.19, 0.1, 10,000)	0.9875	0.0018	51.67	98.33
Original text					
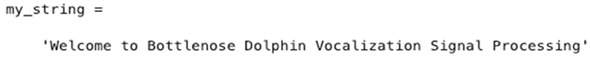					
Correct de-hiding and error decryption text					
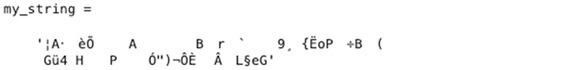					

## Data Availability

The original contributions presented in this study are included in the article. Further inquiries can be directed to the corresponding authors.

## Abbreviations

${SP}_{x}$	Initial point of the x domain for the MCHM
${SP}_{y}$	Initial point of the y domain for the MCHM
α	Bifurcation control parameter for the MCHM
β	Bifurcation control parameter for the MCHM
$n_{I}$	Number of discarded initial MCHM index points
$\delta_{SL}$	Parameter for security level
$L_{F}$	Length of MCHM real-number encryption sequences for the x domain
*MCHMRNES*	Method to generate MCHM real-number encryption sequences
*TIASCIISSBS*	The original text information of the ASCII SS bit streams
*TIASCIISSMCHMEM*	The original text information of the ASCII SS MCHMEM signals
1D	One-dimensional
2D	Two-dimensional
6G	Sixth-generation
ACC	Amplitude correlation coefficient
ASCII	American Standard Code for Information Interchange
BDV	Bottlenose dolphin vocalization
BCUAC	Biologically covert underwater acoustic communication
CC	Covert communication
CGTEHA	Chaotic Gaussian text encryption and hiding algorithm
CUAC	Covert underwater acoustic communication
CRY	Cryptography
DSSS	Direct sequence spread spectrum
DHS	Data-hiding scheme
EEG	Electroencephalography
EH	Encryption and hiding
IH	Information hiding
IoT	Internet of Things
LPD	Low-probability detection
LM	Logistic map
MCHM	Modified chaotic Hénon map
MCHMEM	Modified chaotic Hénon map encryption mechanism
MCHMTIEHM	Modified chaotic Hénon map-based TI encryption and hiding mechanism
MCLSTIEHM	Modified chaotic logistic-sine text information encryption and hiding mechanism
NCBR	Number of changing bit rate
NCCR	Number of changing character rate
NCSSBR	Number of changing SS bit rate
SF	Spread factor
SS	Spread spectrum
TI	Text information
TBDWVs	Taiwan-bottlenose dolphin-whistle vocalizations
UAC	Underwater acoustic communication
UACI	Unified average amplitude change intensity
XOR	Exclusive or
